# On the High Formability of AZ31-0.5Ca Magnesium Alloy

**DOI:** 10.3390/ma11112201

**Published:** 2018-11-07

**Authors:** Umer Masood Chaudry, Tae Hoo Kim, Sang Duck Park, Ye Sik Kim, Kotiba Hamad, Jung-Gu Kim

**Affiliations:** School of Advanced Materials Science & Engineering, Sungkyunkwan University, Suwon 16419, Korea; umer@skku.edu (U.M.C.); eric9707@skku.edu (T.H.K.); chage123@skku.edu (S.D.P.); mgtopia@skku.edu (Y.S.K.)

**Keywords:** AZ31-0.5Ca alloy, microstructure, texture, plastic anisotropy, formability

## Abstract

In this work, we investigated the effect of Ca on the formability of the AZ31 Mg alloy. For this purpose, the microstructure, texture, mechanical properties and formability of AZ31 Mg alloy samples containing 0.5 wt. % Ca (AZ31-0.5Ca) were studied. For comparison, the performance of Ca-Free AZ31 alloy samples with similar grain size was also investigated. In addition, formability of this alloy was reached at a high punch speed. The results of this work showed that the addition of 0.5 wt. % Ca can enhance the formability of the AZ31 alloy, which was three times greater than that of the Ca-Free AZ31 alloy. The improved formability was attributed to the formation of (Mg,Al)_2_Ca particles (~1 μm), which, in turn, contribute to reducing the intensity of the strong basal texture during the primary processing of the alloy. The in-grain misorientation axis analysis determined by electron back-scattered diffraction and critical resolved shear stress calculations carried out by the viscoplastic self-consistent model showed that the non-basal slip systems could be activated in the AZ31-0.5Ca alloy.

## 1. Introduction

For reducing the oil consumption and avoiding the related environmental problems, scientists are always looking for lightweight structural materials that show high performance during both processing and application. Among the various candidates, Mg seems to be the most promising to achieve this target where it is ~33%, 60%, and 75% lighter than Al, Ti, and steel, respectively. On the other hand, the poor ductility and high mechanical anisotropy of Mg limit its potentiality in several fields [1]. One example showing the effect of this limitation on the applicability of Mg was reached a long time ago. In 1935, Bugatti Aerolithe, which is one of the most beautiful and amazing cars ever built, was designed in the Volkswagen Beetle by Jean Bugatti. In this design, Al alloys, which were used to build the car bodies before World War II, were totally replaced by an Mg alloy (Elektron Mg alloy). This alloy had a high strength-to-weight ratio, which can be comparable to those of other materials used for the manufacturing of car bodies. However, the Bugatti Aerolithe was more expensive when compared to the other cars and, ever since, it has remained less usable. The high cost was mainly related to the low formability of the Mg alloy used to build the car where a special interest had to be paid during the processing of different parts. Most of the works reported on the formability and ductility of Mg alloys concluded that the weakening of the basal texture [2] and/or reducing the critical resolved shear stresses (CRSS) of the non-basal slip systems (prismatic and pyramidal) are the key points to enhance the performance of Mg.

Several methods like reducing the grain size during primary processing [3] or alloying with different elements were used to modify the texture and CRSS to improve the formability of Mg alloys. Among these methods, the incorporation of rare earth (RE) elements was found to be a very promising method [4]. However, due to their high cost and incompatibility with recycling constraints, the applicability of Mg alloys containing RE elements is still limited in several fields. Alternative, non-RE elements were used to control the texture evolved after a primary processing and to enhance the formability of Mg alloys. Among the various investigated candidates, Ca showed high potentiality for improving the performance of Mg alloys [5]. In general, the alloying of Ca with pure Mg or Mg alloys can result in stable intermetallic compounds, which, in turn, enhance the grain refinement and texture weakening during the primary processing [6]. Recently, POSCO Mg Inc. has developed an Easy-formable (E-form) Ca-modified AZ31 Mg alloy sheet by a low cost twin roll casting process and succeeded to apply this alloy to the automotive industry [7]. The newly launched Mg alloy (AZ31-0.5Ca Mg alloy) showed improved performance in terms of the room-temperature formability. In order to clarify the main mechanisms responsible for this improved performance, additional studies are still needed. Accordingly, the experiments conducted in the present work were designed to figure out these mechanisms and to give better understanding of the intrinsic effect of Ca in addition to the formability of the AZ31 Mg alloy.

## 2. Methods

### 2.1. Materials and Characterization

The AZ31-0.5Ca alloy sheets with 1-mm thickness used in the present work were provided by POSCO Mg Inc (Pohang, Korea). For comparison, Ca-free AZ31 Mg alloy sheets (~1 mm) with a similar microstructure were also investigated. The composition of the two alloys is shown in Table 1. For microstructure and texture evolution, samples cut from the transverse direction-rolling direction (TD-RD) plane of the sheets were mechanically grinded and polished by using a cross section polisher (Hitachi IM4000, Tokyo, Japan). The polished samples were then examined by using electron back-scattered diffraction (EBSD) in a scanning electron microscope with a field-emission gun (Hitachi S-4300 FESEM, Tokyo, Japan) and the data was analyzed by using TSL OIM 6.1.3 software (V8, EDAX Corporate, Mahwah, NJ, USA). For an accurate measurement, the EBSD scanning was carried out with a small step size (0.05 μm). For texture evolution, the data acquired from the present EBSD experiments were transformed into the orientation distribution function (ODF) utilizing the harmonic series expansion method. These analyses were carried out in a Euler angle space (*φ*_1_  =  0–90°, Φ  =  0–90°, and *φ*_2_  =  0°) by using the non-orthonormal sample symmetry. The measurements of grain size, the misorientation angle, and texture were taken from three different maps including ~2000 grains. Room-temperature tensile tests (ASTM E646-98) were carried out on dog-bone specimens cut from the sheets of the two alloys (AZ31 and AZ31-0.5Ca) along three different directions including RD, 45° from RD and TD. Mechanical anisotropy of the alloys was investigated by analyzing the deformation behavior of the samples at 10% tensile strain. Accordingly, the Lankford values (*r-value*), the average *r-value* (*r_avg_*), and the planar anisotropy (Δ*r*) were determined by using the following equations.

(1) $$\;r=\frac{\varepsilon_{w\;}}{\varepsilon_{t}}\;$$

(2) $$\;r_{ave}=\frac{1}{4}\;\left|r_{RD}+r_{TD}+2r_{45}\right|\;$$

(3) $$\;\Delta r=\frac{1}{2}\;\left|r_{RD}+r_{TD}-2r_{45}\right|\;$$

where *ε_w_* and *ε_t_* are the true width and thickness strains at 10% tensile deformation, respectively, and *r_RD_*, *r*_45_, and *r_TD_* are *r-value*s are along the three different directions. Erichsen cupping tests were conducted on 90 mm × 90 mm sheets at room temperature using two different punch speeds (0.33 mm/s and 0.08 mm/s). The results of five tests were averaged to obtain the Erichsen value of each alloy. The conditions of Erichsen tests are presented in Table 2.

### 2.2. VPSC Modeling

The viscoplastic self-consistent (VPSC) model was employed to simulate the texture that evolved from the two alloys (AZ31 and AZ31-0.5Ca) after 10% tensile deformation starting from 2000 discrete orientations taken from EBSD results of the non-deformed alloys (Figure 3). This occurred to predict the activity of various slip systems during the deformation (basal and non-basal slip systems) [7]. In this regard, the Voce hardening law (Equation (4)) was introduced in this model to calculate the CRSS of the slip systems. Accordingly, by increasing the CRSS of the *s*th system (*τ^s^*) as a function of the total shear strain (Γ) accumulated in the grain, the hardening response of individual slip and twinning systems can be modeled by using the equation below.

(4) $$\;\tau^{s}=\;\tau_{0}^{s}+\left(\tau_{1}^{s}+\;\theta_{1}^{s}\;\Gamma \right)\left(\;1-\exp\left(-\Gamma \;\left|\frac{\theta_{0}^{s}}{\tau_{1}^{s}}\right|\right)\right)\;$$

where *s*, *θ*_0_^s^, *θ*_1_*^s^*, *τ*_0_*^s^,* and (*τ*_0_*^s^ + τ*_1_*^s^*) are the slip system, initial and final slopes of the hardening curve, initial critical resolved shear stress, and the back-extrapolated CRSS, respectively. In this work, only tension twins were considered for modeling because other twining systems such as compressive and double twins were unobservable after the 10% tensile deformation. 

## 3. Results

### 3.1. Formability

**Figure 1:**
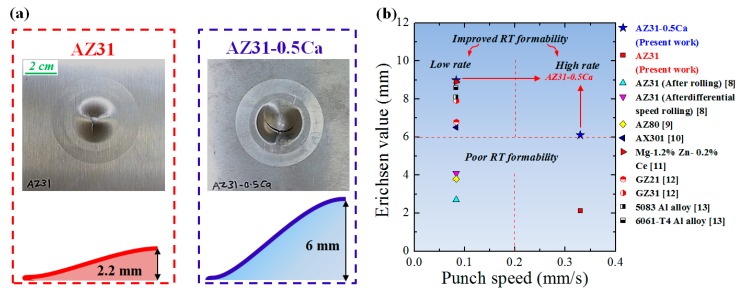
(**a**) Side views taken from the AZ31 and AZ31-0.5Ca alloy sheets after Erichsen test; (**b**) Comparison between Erichsen values of the AZ31-0.5Ca alloy studied in the present work with some other Mg and Al alloys.

Figure 1a shows the cups left after the room-temperature Erichsen tests were carried out on the AZ31 and AZ31-0.5Ca sheets at a punch speed of 0.33 mm/s. The addition of 0.5 wt. % Ca significantly enhanced the formability of the AZ31 alloy where Erichsen values of ~6 mm and 9 mm were recorded for the AZ31-0.5Ca alloy sheets and tested at punch speeds of 0.33 mm/s and 0.088 mm/s, respectively. The Erichsen value of this alloy at 0.33 mm/s is about three times higher than the value recorded for the AZ31 alloy at the same speed. For comparison, the Erichsen values recorded for several Mg and Al alloys are presented in Figure 1b as a function to the punch speed [8,9,10,11,12,13]. Generally, due to the high hardening occurred at high punch speeds [10,11,12,13], low speeds (<0.09 mm/s) are usually used in order to increase the formability limit of metallic sheets. As shown by Figure 1b, the AZ31-0.5Ca alloy investigated in the present work exhibited higher formability than other Mg alloys [8,9,10,11] and, more importantly, the improved formability was reached at a high punch speed (0.33 mm/s). The Ca addition, accordingly, resulted in a high-rate-improved formability, which can be reached at room temperature. To understand the effect of the Ca addition on the formability of the AZ31 Mg alloy, the microstructure evolution, texture, and related mechanical properties of AZ31 and AZ31-0.5Ca alloys were investigated and discussed in the following sections.

### 3.2. Microstructure and Texture

**Figure 2:**
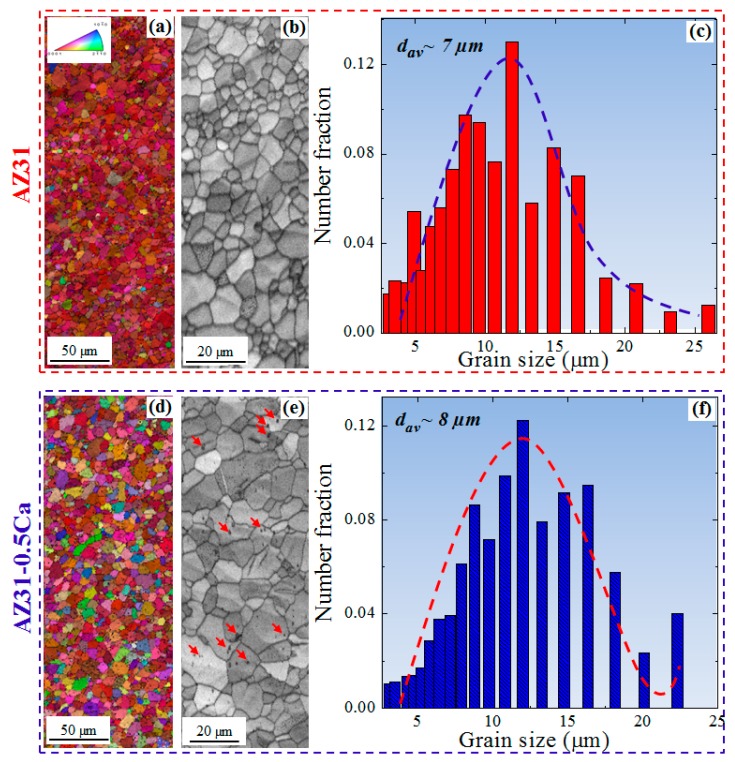
(**a**,**d**) ND inverse pole figure maps; (**b**,**e**) image quality maps; and (**c**,**f**) grain size distributions of the AZ31 and AZ31-0.5Ca alloys, respectively. The grain size measurements by EBSD experiment were conducted at a tolerance angle of 2°.

**Figure 3:**
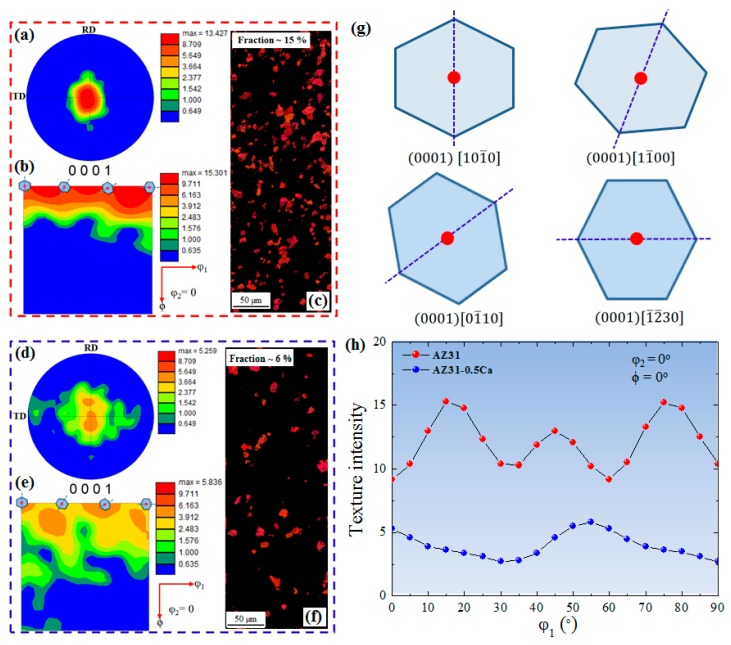
(**a**,**d**) Pole figures; (**b**,**e**) orientation distribution function; and (**c**,**f**) ND inverse pole figure maps of partitioned basal grains of the AZ31 and AZ31-0.5Ca alloys, respectively; (**g**) The main orientations of the basal texture; (**h**) Orientation distribution function intensity of the two alloys along *φ*_1_ (*φ*_2_  =  0°, Φ  =  0°).

**Figure 4:**
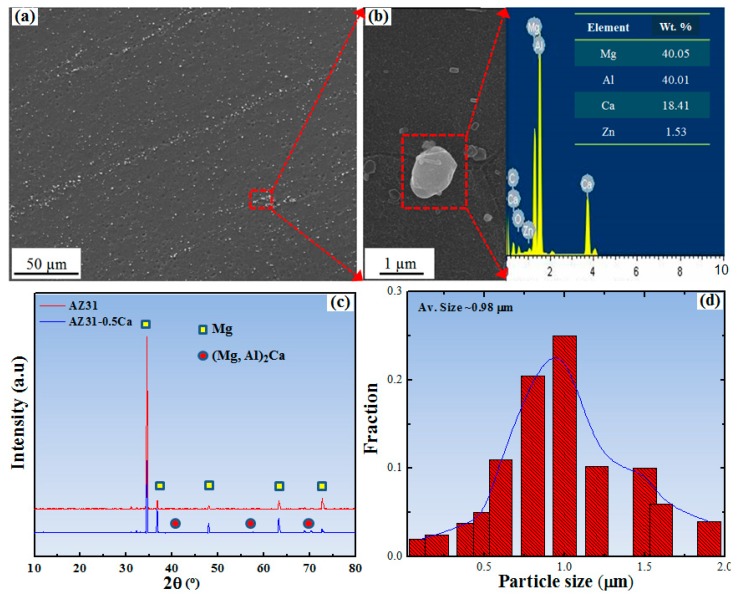
(**a**,**b**) SEM micrographs of the AZ31-0.5Ca alloy showing fine (Mg,Al)_2_Ca particles and the related energy-dispersive X-ray spectroscopy taken on the particles [7]; (**c**) X-ray diffraction pattern of AZ31 and AZ31-0.5Ca; (**d**) (Mg,Al)_2_Ca particle size distributions in AZ31-0.5Ca.

Figure 2 shows the electron back-scattered diffraction (EBSD) data including the normal direction (ND) inverse pole figure (IPF) maps, image quality (IQ) maps, and grain size distribution of AZ31 and AZ31-0.5Ca alloys. It is clearly evident that both alloys have similar microstructural features including grain size and morphology (Figure 2a,d), which can also be confirmed from grain size distribution of both alloys (Figure 2c,f). On the other hand, two main variations can be noted, which is that the former is the higher number of randomly-oriented grains in the AZ31-0.5Ca alloy, as shown by the IPF maps (Figure 2a,d), and the latter is the presence of fine particles in the AZ31-0.5Ca alloy, which is shown by the low quality area in the IQ map in Figure 2e (indicated by red arrows). In the AZ31-0.5Ca alloy, randomly-oriented grains, which are those with the *c-axis* tilted away from the ND of the sample, indicate the weak basal texture evolved in this alloy. On the other hand, the initial texture of the AZ31 alloy investigated in the present work is characterized by more grains with basal orientations such as $\left(0001\right)\left[10\bar{1}0\right]$, $\left(0001\right)\left[1\bar{1}00\right]$, $\left(0001\right)\left[0\bar{1}10\right],$ and $\left(0001\right)\left[\bar{1}\bar{2}30\right]$. This can be clarified by (0001) pole figures (PF) and the orientation distribution function (ODF) maps of the AZ31 and AZ31-0.5Ca alloys obtained by EBSD, which is shown in Figure 3a,b,d,e, respectively. (0001) PFs of the alloys in Figure 3a,d show that the AZ31 alloy has a higher maximum intensity (~13) as compared to the AZ31-0.5Ca alloy (~5) with basal poles broadly distributed along the transverse direction (TD) of the sample. Reduced Euler space (*φ*_2_: 0°, *φ*_1_: 0–90°, Φ: 0–90°) ODF maps presented in Figure 3b,e show the basal texture components $(\left(0001\right)\left[10\bar{1}0\right]$, $\left(0001\right)\left[1\bar{1}00\right]$, $\left(0001\right)\left[0\bar{1}10\right]$, and $\left(0001\right)\left[\bar{1}\bar{2}30\right]$ (Figure 3g)) along *φ*_1_ (0–90°) and the intensities of these components are weaker for AZ31-0.5Ca as compared to the AZ31 alloy in Figure 3b,e,h. Additionally, the partitioned maps of the basal-oriented grains (Figure 3c,f) show the evolution of fewer numbers of basal-oriented grains in the AZ31-0.5Ca (6%) as compared to the AZ31 alloy (15%). For the partitioning of basal-orientated grains, a tolerance angle of 5° was used. Coming back to the above-mentioned variations, the fine particles observed in the IQ map of the AZ31-0.5Ca alloy are analyzed further by using scanning electron microscopy with energy dispersive spectroscopy (SEM/EDS). Figure 4a–c shows low and high magnifications SEM micrographs, the related EDS analysis, and the X-ray diffraction pattern of the two alloys. For the particle size distribution presented in Figure 4d, almost 200 particles distributed within the grains and on the grain boundaries were selected from different positions. The selection of these particles was carried out based on their composition (Figure 4b). The EDS measurements of these particles revealed that they are mainly composed of Mg ~40 wt. %, Al ~40 wt. %, and Ca ~18 wt. % (Figure 4b). The present SEM/EDS data is consistent with the predication work carried out by Grobner and Schmid-Fetzer [14], which indicates that the addition of Ca to the AZ31 alloy can lead to the formation of an (Mg,Al)_2_Ca intermetallic compound [15]. In addition, this is consistent with the XRD patterns of the two alloys (Figure 4c) where new peaks related to (Mg,Al)_2_Ca appeared in the AZ31-0.5Ca alloy [16]. The presence of such particles might induce the evolution of specific dynamic and static recrystallization behaviors during the primary processing of this alloy [17]. Based on this point, additional experimentation is still needed in order to clarify how these particles control the recrystallization behaviors of the AZ31-0.5Ca alloy.

### 3.3. Mechanical Performance

**Figure 5:**
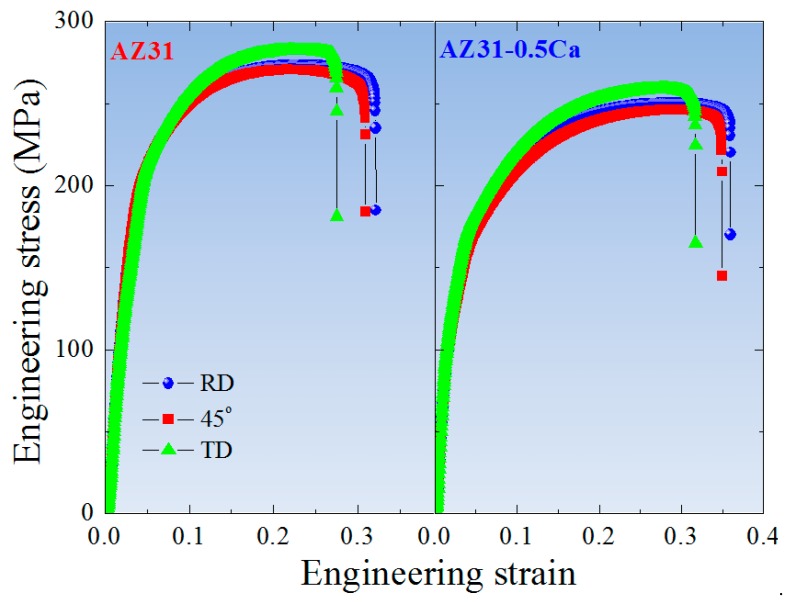
Room temperature engineering stress-engineering strain curves of the AZ31 and AZ31-0.5Ca alloys along RD, 45° from RD, and TD.

Figure 5 shows room-temperature tensile curves of the AZ31 and AZ31-0.5Ca alloys tested along three different directions, which includes RD, 45° from RD, and TD. The tensile data including yield strength (YS), ultimate tensile strength (UTS), uniform elongation (UE), total elongation (TE), and the strain hardening exponent (*n*) are listed in Table 3. Strain hardening exponents presented in Table 3 were calculated by using true stress-true strain curves of the alloys (not shown here). Generally, along the three directions, the AZ31-0.5Ca alloy is more ductile and weaker than the AZ31. For both alloys, AZ31 and AZ31-0.5Ca, the YS was the highest along RD, which was followed by TD and 45°, respectively. In addition, the two alloys show identical behaviors in which the TE was the highest along the RD followed by 45° and TD. In order to clarify the effect of the composition and the related texture on the mechanical anisotropy, Lankford values (*r*-*value*), the average *r-value* (*r_avg_*), and planar anisotropy (Δ*r*) of the alloys were determined and compared (Table 3). Although the two alloys nearly showed the same trend along the various directions, they have distinct mechanical anisotropy and strain hardening capabilities. With *r*-*values* and Δ*r* close to 1 and 0, respectively, the AZ31-0.5Ca alloy exhibits a lower plastic anisotropy when compared to AZ31, as shown in Table 3. In addition, the strain hardening exponents of this alloy (AZ31-0.5Ca) along the three different directions are higher than those of the AZ31 alloy. The high strain hardening capability of the AZ31-0.5Ca alloy reduces the mechanical instabilities during plastic deformation and, hence, enhances the uniform ductility (shown in Table 3).

## 4. Discussion

**Figure 6:**
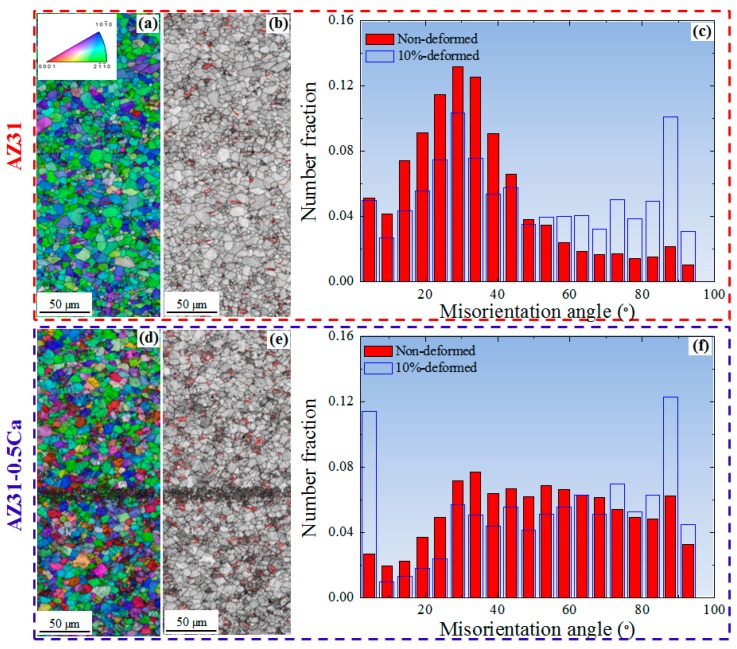
(**a**,**d**) ND inverse pole figure maps; (**b**,**e**) image quality maps; and (**c**,**f**) misorientation angle distributions of the 10%-deformed AZ31 and AZ31-0.5Ca alloy, respectively. For comparison, the misorientation angle distributions of the initial samples (non-deformed) were also included. In addition, the tension twin boundaries (~86°) were shown by the red lines in the image quality maps (**b**,**e**).

**Figure 7:**
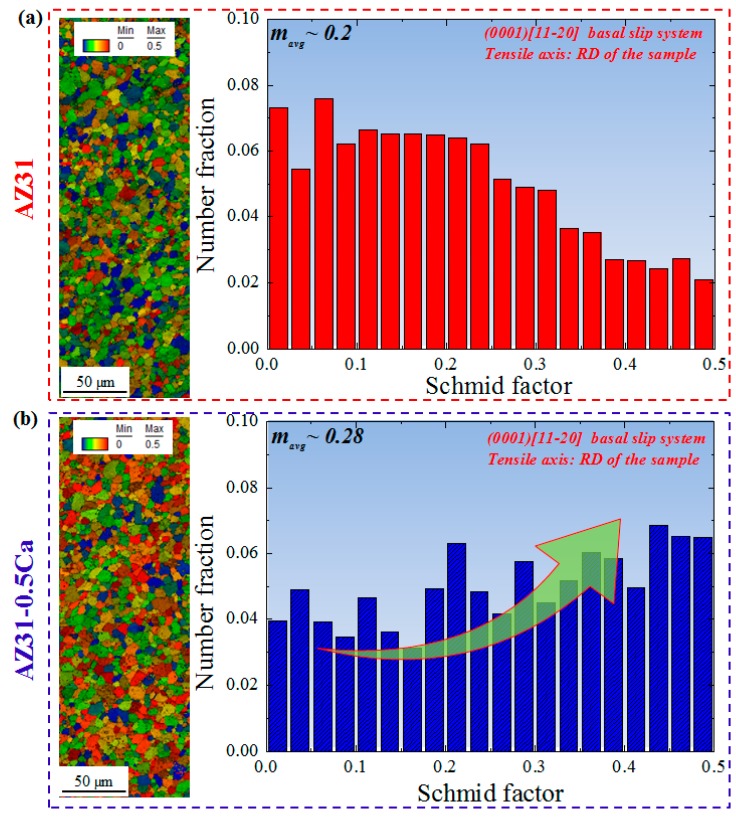
(**a**,**b**) Schmid factor maps and Schmid factor distribution of the AZ31 and AZ31-0.5Ca alloys, respectively. The Schmid factor was determined for the $\left(0001\right)\left[11\bar{2}0\right]$ basal slip system along the RD as the tensile axis.

To figure out the improved formability recorded for the AZ31-0.5Ca alloy, the microstructure and texture evolution of the two alloys after a 10% tensile deformation were investigated. Figure 6 shows the ND-IPF maps, IQ maps, and misorientation angle distributions of the two alloys tensile-deformed at 10% along the RD. For the comparison, the misorientation angle distributions of the non-deformed alloys were also included. It is clearly seen that both alloys show an evolution of boundaries, which are related to tension twins ($\langle \bar{1}011\rangle$ $\left\{10\bar{1}2\right\}$ ~86°) after the tensile deformation. This was indicated by the red boundaries in the IQ maps (Figure 6b,e). The fraction of these boundaries is a little bit higher in the AZ31-0.5Ca alloy sample (~12%) when compared to AZ31 (~10%) (Figure 6c,f). Tension twins, which act as a secondary deformation mechanism beside the basal slip, usually accommodate the extension along the *c-axis*. In addition, they can easily form in grains with a high Schmid factor and grow thickly to overtake the parent grains, which results in high ductility. Usually, 30% to 40% tension twins evolve after the 10% tensile deformation of weak-basal-textured pure Mg and its alloys [18]. In the present work, the low fraction of the tension twins observed for the AZ31 alloy (~10%) is attributed to its strong basal texture, which is shown by Figure 3 and by the Schmid factor maps for the basal slip (Figure 7). On the other hand, the 10% tensile deformation of the AZ31-0.5Ca alloy with a weak basal texture leads to a lower fraction of tension twins (~12%) when compared to the fraction evolved in the weak-basal-textured Mg materials (30–40%) after the same amount of deformation. This, in turn, confirms a possible contribution of non-basal slip to the deformation of the AZ31-0.5Ca alloy. To characterize the type of non-basal slip, which might be activated, the distribution of in-grain misorientation axes (IGMA) were determined for the deformed alloys [19]. IGMA analyses are used to identify the slip modes throughout a misorientation range between 2.5° and 5° where, in this range, the in-grain misorientations are mainly caused by the activation of slip systems [19]. An activation of a single slip in a crystal was considered in order to explain the relationship between the activated slip system and the IGMA. In general, the presence of dislocations can bend the crystal, which leads to a slight in-grain misorientation (2.5°–5°) [19]. The in-grain misorientation due to a single slip can be described by the following equation.

**Figure 8:**
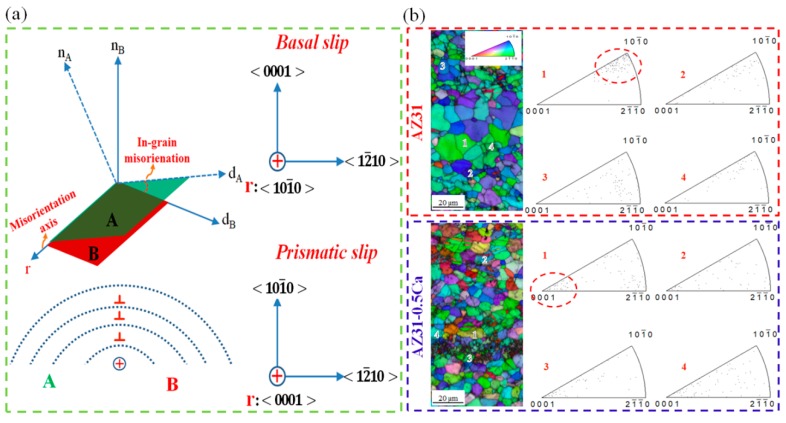
(**a**) Schematics showing the main concept of the relation between the in-grain misorientation axis and the activated slip system; (**b**) ND inverse pole figure maps and related in-grain misorientation axis analysis taken for four randomly-selected grains from the AZ31 and AZ31-0.5Ca alloys. The two examples indicated by the dashed circles (grains 1 in the AZ31 and AZ31-0.5Ca alloys) show the shifting of the misorientation axis toward <0001> in the AZ31-0.5Ca and toward $\langle 2\bar{1}\bar{1}0\rangle –\langle 10\bar{1}0\rangle$ in AZ31.

(5)  r(s)=n(s)×d(s) 

where *s* is the slip system in which the dislocation is generated, *r* is the Taylor axis around which the crystal is bent, *n* is the axis of the slip plane, and *d* is the slip direction. According to this equation, the characteristics of the crystal bending due to the single slip are mainly associated with the type of the slip. This can be shown by Figure 8a where two types of pure edge dislocations are considered including the <a> basal dislocation and the <a> prismatic dislocation. Based on Figure 8a, misorientation axes close to the $\langle 10\bar{1}0\rangle$ indicate a high activity of the <a> basal slip while the <a> prismatic slip is indicated by misorientation axes close to 〈0001〉. Figure 8b shows IGMA analyses of four different grains taken from the tensile-deformed AZ31 and AZ31-0.5Ca samples. The results clearly show that, after the tensile deformation, the distribution of IGMA of the grains selected from the AZ31 alloy is concentrated between $\langle 10\bar{1}0\rangle$ and $\langle 2\bar{1}\bar{1}0\rangle \;$, which indicates that the basal slip is the dominated deformation mode in this alloy. On the other hand, the distribution of IGMA of the grains selected from the AZ31-0.5Ca alloy are distributed around 〈0001〉, which suggests that the prismatic slip is absolutely activated [20,21,22,23].

Several studies reported the effect of the composition on the activity of slip systems for various Mg alloys and related performance of these alloys. For example, Yuasa et al. [24] studied the effect of Ca addition (~0.06 wt. %) on the formability and associated slip modes in the Mg-1.5Zn alloy. Their results showed that the improved formability of this composition (Mg-1.5Zn-0.06Ca) was essentially attributed for the activation of the prismatic slip, which, in turn, is related to the role of Ca solutes in reducing the generalized stacking fault energy of this system. In the present work, however, a higher amount of Ca (0.5 wt. %) was alloyed, which was above the solubility limit of Ca in Mg [25] and this addition resulted in the formation of the intermetallic compound, as shown by the EDS/SEM analysis (Figure 4). Accordingly, the effect of the 0.5wt. % Ca addition on the activity of the prismatic slip cannot be figured out based on the reduced generalized stacking fault energy.

As an alternative, the activation of the non-basal slip systems might be induced by changing the relative CRSSs (CRSS_non-Basal_/CRSS_Basal_) [26]. As compared to single-crystal Mg alloys, CRSSs of polycrystalline Mg alloys are related to the hardening contributions by grain boundaries, dislocations, and second-phase particles. Accordingly, for a polycrystalline Mg alloy, the actual value of CRSS (equivalent to that recorded at the yielding of the single-crystal Mg alloy) is given by the equation below.

(6)  τ=CRSS+Δτ 

where *τ* is the effective CRSS value obtained in a hardened state of the polycrystalline Mg alloy and Δ*τ* is the shear stress related to the hardening contributions. For the single-crystal Mg alloys, Δ*τ* is equal to zero and, hence, the CRSS of the basal slips (CRSS_B_) and of prismatic slips (CRSS_P_) are found to be [27] the following values below.

(7) $$\;{\mathrm{CRSS}}_{B}\sim 1.0\;\mathrm{MPa}\;$$

(8) $$\;{\mathrm{CRSS}}_{P}\sim 40\;\mathrm{MPa}\;$$

Based on Equation 6 and the CRSS values of the basal and non-basal slip systems reported for the single-crystal Mg alloys [27], the following equation can be written for polycrystalline Mg alloys.

(9) $$\;\frac{\tau_{P}}{\tau_{B}}=\frac{40+\Delta \tau }{1+\Delta \tau }\;$$

According to this equation, the activity of the prismatic slip would be controlled through the shear stress raised from the hardening effect of the microstructure (Δ*τ*). In our work, the second-phase particles ((Mg,Al)_2_Ca) formed due to the Ca addition seem to have the effective hardening factor during plastic deformation of this alloy when compared to the AZ31 alloy.

**Figure 9:**
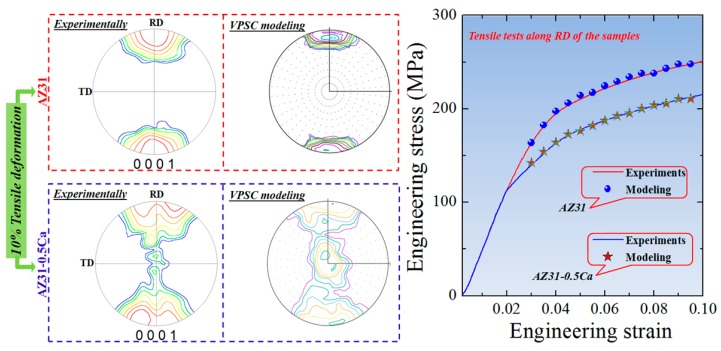
Experimentally observed and VPSC-modeled (0001) pole figures and tensile curves of the 10%-tensile-deformed AZ31 and AZ31-0.5Ca alloy samples. For tensile curves, the samples of the two alloys were deformed along the RD.

**Figure 10:**
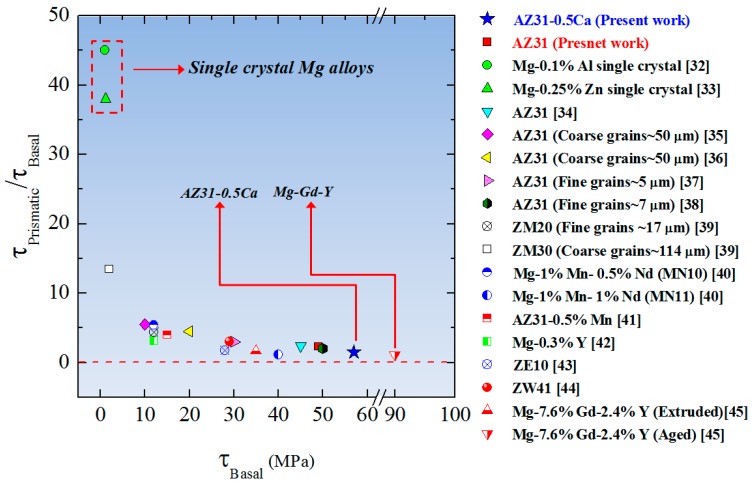
The ratios between the effective CRSSs (*τ*_Prismatic_/*τ*_Basal_) as a function to the effective CRSS of the basal slip for several Mg alloys (single-crystal and polycrystalline) [7]. The CRSSs presented in this figure were determined by using various models including the viscoplastic self-consistent (VPSC) model, the elastoplastic self-consistent (EPSC) model, the elastic viscoplastic self-consistent (EVPSC) model, and the Sachs model.

In order to confirm the above-discussed idea, the CRSS values of the various slip systems of the alloys (AZ31 and AZ31-0.5Ca) were calculated by using polycrystalline plasticity simulation based on the viscoplastic self-consistent (VPSC) model [28]. Based on 2000 orientations collected from the initial texture of two alloys (Figure 3), the VPSC model was used to simulate the texture until 10% tensile deformation. The voce hardening law and its parameters were adjusted until the 10% simulated texture and stress-strain curves matched those experimentally obtained (Figure 9) [29]. The results obtained by the VPSC modeling (Table 4) reveal that the shear stress needed for plastic deformation of the present alloys is greater than that needed for single crystals. This is due to the hardening imposed by the microstructure (grain boundaries effect), which is shown by Equations (6)–(8). More importantly, the CRSS of the basal slip in the AZ31-0.5Ca alloy was higher than that of the AZ31 alloy (49 vs. 57 MPa). The further hardening recorded for the AZ31-0.5Ca is attributed to the Ca addition and the formation of the intermetallic compound ((Mg,Al)_2_Ca), which might impede the basal slip. In addition, the relative CRSSs (*τ*_Prismatic_/*τ*_Basal_) of the AZ31-0.5Ca alloy was less than that of the AZ31 (1.5 vs. 2.2). This is consistent with previous works, which reported that the ratios between the effective CRSSs (*τ*_Prismatic_/*τ*_Basal_) in Mg alloys get closer to the unity when the hardening induced by the microstructure is high [30]. Such behavior leads to an increase of the activity of the prismatic slip, which can enhance formability. In this case, upon the activation of the prismatic slip, not only is the plasticity enhanced but also the plastic anisotropy is reduced [31]. The alternation of the *τ*_Prismatic_/*τ*_Basal_ ratio in Mg alloys by strengthening the basal slip has been reported in several works, which is shown in Figure 10 [32,33,34,35,36,37,38,39,40,41,42,43,44,45]. As compared to the highly formable Mg alloy containing RE elements [40], the present Mg alloy (AZ31-0.5Ca) exhibits a reasonable reduced ratio of 1.5 vs. 1.1 in Mg-7.6% Gd-2.4% Y [45].

In summary, the present work showed that the modification of the well-known Mg alloy, AZ31, by 0.5 wt. % Ca could lead to a highly formable Mg-based material capable of withstanding a high punch speed at room temperature. This improved formability of the AZ31-0.5Ca alloy was attributed to the role of the Ca addition in weakening the basal texture during the primary processing. This, in turn, leads to a low plastic anisotropy, which is shown by the three-direction tensile tests. In addition, the IGMA analysis carried out by EBSD on 10%-tensile-deformed samples revealed the AZ31-0.5Ca alloy, which exhibited a higher activity of the non-basal slip (prismatic slip) during the deformation as compared to the Ca-free alloy (AZ31). The modeling by VPSC confirmed that the high activity of such slip systems in the AZ31-0.5Ca alloy is due to the reduced relative CRSSs (*τ*_Prismatic_/*τ*_Basal_). All in all, the formation of (Mg,Al)_2_Ca due to the addition of 0.5 wt. % Ca can weaken the basal texture during the primary processing and increase the activity of the non-basal slips. This leads to low plastic anisotropy, enhanced plasticity, and, as a result, improved formability. Additional experiments are still needed in order to investigate the effect of (Mg,Al)_2_Ca particles on the static and dynamic recrystallization behaviors of the AZ31-0.5Ca alloy.

## Figures and Tables

**Table 1:** The composition of AZ31 and AZ31-0.5Ca alloys.

Alloy	Al	Zn	Ca	Si	Mn	Fe	Cu	Mg
AZ31	2.99	0.73	-	0.024	0.20	0.0026	0.0003	Bal.
AZ31-0.5Ca	3.12	0.76	0.5	0.023	0.31	0.0042	0.0012	Bal.

**Table 2:** The conditions of the Erichsen test used in the present work.

Load (kN)	Punch Size (mm)	Punch Speed (mm/s)	Specimen Size (mm)	Specification
350	20	0.33	90 × 90	ISO 20482

**Table 3:** Room-temperature tensile properties and mechanical anisotropy parameters of AZ31 and AZ31-0.5Ca alloys. Strain hardening exponents presented in Table 3 were calculated by using true stress-true strain curves of the alloys (not shown here).

Alloy	Tensile Direction	YS (MPa)	UTS (MPa)	UE (%)	TE (%)	*n*	*r*	*r_avg_*	Δ*r*
AZ31	RD	176	275	24	32	0.2	2.34	2.3	0.72
	45°	161	272	22	29	0.17	2.7		
	TD	171	283	23	27	0.21	1.62		
AZ31-0.5Ca	RD	147	248	31	38	0.28	0.71	0.6	0.16
	45°	125	237	30	35	0.27	0.52		
	TD	139	251	30	32	0.26	0.69		

**Table 4:** CRSS values and hardening parameters of the AZ31 and AZ31-0.5Ca alloys as modeled by the VPSC. All values listed in this table were obtained for tensile tests along the RD of the samples.

Alloy	Deformation Mode	*τ*_0_	*τ*_1_	*θ*_0_	*θ*_1_
AZ31	<a> basal slip	49	8	122	35
	<a> prismatic slip	108	31	155	11
	<a + c> pyramidal slip	311	76	88	18
	Tension twin	6	97	109	25
AZ31-0.5Ca	<a> basal slip	57	9	115	62
	<a> prismatic slip	91	37	148	8
	<a + c> pyramidal slip	305	44	64	22
	Tension twin	15	185	110	55
